# Impact of Constipation on Health Outcomes in Medically Hospitalized Patients: A Prospective Study on Laxative Use and Health Care Outcomes

**DOI:** 10.3390/medicina60101599

**Published:** 2024-09-29

**Authors:** Abdullah M. Al Alawi, Jawahar Al Nou’mani, Nahid Al Abri, Maryam Al Sabbri, Juhaina Salim Al-Maqbali

**Affiliations:** 1 Department of Medicine, Sultan Qaboos University Hospital, Muscat 123, Oman; dr.abdullahalalawi@gmail.com; 2 Internal Medicine Residency Training Program, Oman Medical Specialty Board, Muscat 130, Oman; 3 Collage of Medicine and Health Science, Sultan Qaboos University, Muscat 123, Oman; nahidalabri461@gmail.com (N.A.A.); msabbari98@gmail.com (M.A.S.); 4 Department of Pharmacy, Sultan Qaboos University Hospital, Muscat 123, Oman; juhaina@squ.edu.om; 5 Department of Pharmacology and Clinical Pharmacy, Sultan Qaboos University, Muscat 123, Oman

**Keywords:** constipation, inpatient, in-hospital, laxatives, mortality

## Abstract

*Background and Objectives:* Constipation affects health-related quality of life and increases hospital visits. We conducted this prospective cohort study to assess laxative use, health outcomes of constipation in medically hospitalized patients, and related health outcomes. *Materials and Methods:* A prospective single-center study included all adult patients admitted under the General Internal Medicine Unit from 1 February 2022, to 31 August 2022. Constipation was defined using the Constipation Assessment Scale (CAS). Patients were assessed for 28 days during their hospital stay and up to 90 days post-discharge. *Result:* Among the included patients, 62.45% experienced constipation, which was associated with poor health outcomes including delirium (*p* = 0.048), intensive care admission (*p* < 0.01), cardiopulmonary arrest (*p* < 0.01), inpatient mortality (*p* < 0.01), longer hospital stay (*p* < 0.01), 90-day mortality (*p* < 0.01), and 90-day hospital readmission (*p* < 0.01). Laxative treatment was administered to only 33.93% of patients with constipation and was more commonly used among older patients (*p* < 0.01), those with high CAS scores (*p* < 0.01), longer hospital stays (*p* < 0.01), and critically ill patients (intensive care admission) (*p* = 0.01), as well as those who had cardiopulmonary arrest (*p* < 0.01) and high inpatient mortality (*p* < 0.01). *Conclusions:* This study identified several associations between constipation and poor health outcomes and highlighted the underutilization of laxatives in treating constipation. It is vital to interpret our results with caution. Therefore, we believe that a randomized controlled trial will help enhance our understanding of the interaction between constipation, laxative use, and poor health outcomes.

## 1. Introduction

Constipation is a prevalent gastrointestinal condition that significantly impacts individuals and healthcare systems, decreasing health-related quality of life and elevating healthcare costs [1,2]. The reported prevalence of constipation among adults in community settings varies widely, ranging from 2% to 35%, attributed to differences in age groups, culture, diet, environment, and variations in defining chronic constipation [3,4,5]. Among acutely hospitalized patients, the reported prevalence of constipation varies, ranging from 14.8% to 65% [6,7], and a recent prospective study, which utilized a well-validated constipation score, reported a constipation prevalence of 55.6% among medically hospitalized patients [4]. Several factors were identified to be associated with constipation among hospitalized patients, including old age, frailty, the use of certain medications like calcium channel blockers and opioids, specific diagnoses such as heart failure, reduced mobility, prolonged hospital stays, and conditions like dyssynergic defecation and inadequate bowel movement propulsion [4,5,8,9].

Constipation increases the risk of developing hemorrhoids, anal fissures, urinary retention, rectal prolapse, volvulus, megacolon, self-reported depression, and other psychologic symptoms [2,10,11,12,13,14]. In addition, constipation shows a positive association with myocardial infarction, stroke, and overall cardiovascular events [15,16]. While the exact mechanism remains speculative, potential links include constipation promoting gut dysbiosis, contributing to systemic inflammation and cardiovascular risk. Moreover, straining during constipation may acutely increase blood pressure, potentially triggering cardiovascular events [15].

Among intensive care unit (ICU) hospitalized patients, those with constipation had a notably prolonged adjusted length of hospital stay (LOS) compared to those without constipation, leading to elevated inpatient hospital costs. Various complications associated with constipation, such as peritonitis, fecal impaction, and bowel obstruction, were identified as factors contributing to increased mortality rates [17]. Another study has shown that constipation was associated with increased mortality and hospital-associated infections among patients admitted to ICU [18]. Among patients hospitalized with acute stroke, new-onset constipation was associated with poor health outcomes at 12 weeks [19]. There are very few studies that address adverse health outcomes related to constipation in medically hospitalized patients in general wards [7].

In light of the insufficient high-quality evidence regarding constipation in medically hospitalized patients within general wards, we undertook this prospective cohort study to investigate laxative use and the short- and long-term health outcomes related to constipation within this demographic. This present study presents follow-up findings from a previously published study entitled “Prevalence, Recognition, and Risk Factors of Constipation among Medically Hospitalized Patients: A Cohort Prospective Study” [4].

## 2. Methods & Materials

### 2.1. Study Setting

The study was conducted at Sultan Qaboos University Hospital (SQUH), a major academic center known for providing high-quality, specialized care to patients referred from across the Sultanate of Oman [20]. This study presents follow-up outcomes for adult patients (≥18 years) who were previously included in a previously published paper, with detailed methods outlined [4].

### 2.2. Sample Size

The reported prevalence of constipation in acutely hospitalized patients in our population was 55.6% [4], reported inpatient mortality is around 5% [7], and the rate of hospital readmission is around 20% [21]. Hence we estimated that we need at least 250 patients to study inpatient mortality and hospital readmission in patients with constipation.

### 2.3. Study Design and Population

This prospective cohort study included all adult patients (≥18 years) admitted under the care of the General Internal Medicine (GIM) unit at Sultan Qaboos University Hospital (SQUH) from 1 February, 2022, to 31 August, 2022. The inclusion criteria consisted of patients aged 18 years and above. However, patients who were directly admitted to acute care units, such as the Intensive Care Unit (ICU) or High Dependency Unit (HDU), as well as patients with acute abdomen, were excluded from the study [4].

### 2.4. Data Collection

We collected comprehensive data, including demographic profiles, comorbidity information, admission details (length of stay and primary diagnosis), and data relating to constipation (including prevalence, severity, and therapeutic interventions). Additionally, we gathered data on adverse hospital outcomes, such as hospital readmission, inpatient cardiopulmonary arrest, transfers to acute care units, and inpatient mortality. The follow-up data were acquired through a review of the hospital record system and complemented by telephone-based follow-ups.

Constipation was identified by trained research assistants using a well-validated score, which is the Constipation Assessment Scale (CAS). The CAS total scores span from 0 to 16. A score of 0 to 1 signifies the absence of constipation, while a score of 2 to 6 indicates mild to moderate constipation. A score within the range of 7 to 16 is indicative of severe constipation [22]. We have previously reported detailed descriptions of our methods, including the precise definitions and assessment criteria applied to constipation within our study cohort [4].

### 2.5. Statistical Analysis

The normality of continuous variables was assessed using the Shapiro–Wilk test. Normally distributed data were presented as mean and standard deviation (SD), while non-normally distributed data were expressed as median and interquartile range (IQR). Group differences were assessed using one-way ANOVA for normally distributed variables and the Kruskal–Wallis rank test for non-normally distributed ones. Categorical variables were summarized with counts and percentages, and group differences were examined through Pearson’s χ^2^ tests or Fisher’s, as appropriate. Binary logistic regression analysis incorporated relevant data, such as age, gender, comorbidities, frailty score, length of hospital stay, and constipation-related information, to assess the predictive value of constipation or laxative use for inpatient mortality, cardiopulmonary arrest, 90-day hospital readmission, and 90-day mortality. Two-sided *p*-values below 0.05 were considered statistically significant. Stata v. 18.0 software package (StataCorp LLC. College Station, TX, USA) conducted our statistical analysis.

## 3. Results

Among all the screened patients, 538 met the inclusion criteria. There were 267 females (49.63%), and the median age was 59 (IQR: 42–72) years. Hypertension (52.42%) and diabetes mellitus (41.82%) were the most common comorbidities. Additionally, anemia was prevalent among this cohort (50.74%). Regarding the primary diagnosis, diseases of the circulatory system (17.47%) and diseases of the respiratory system (16.36%) were the most common diagnoses. A detailed description of the cohort, including the primary diagnoses, is presented in Table 1.

Among the 538 included patients, 306 (56.88%) had constipation present upon admission, while 30 other patients (5.58%) developed constipation during hospitalization. The cumulative incidence of constipation was 62.45% (*n* = 336). The majority of patients (49.07%, *n* = 264) had mild to moderate constipation according to the CAS, with osmotic laxatives being the most commonly used laxatives. Table 2 provides a detailed summary of incidence of constipation and constipation severity.

As shown in Table 3, regarding health outcomes, 36 patients (6.69%) were transferred to the ICU or HDU, 31 patients (5.92%) experienced cardiopulmonary arrest, and 30 patients (5.58%) died during the same hospitalization. Severe constipation was associated with increased delirium (11.11% vs. 5.3% vs. 3.47%; *p* = 0.048), prolonged length of hospital stay (6 vs. 5 vs. 3 days; *p* < 0.01), a higher requirement for ICU or HDU admissions (16.67% vs. 6.06% vs. 3.96%; *p* < 0.01), increased cardiopulmonary arrest (15.71% vs. 5.86% vs. 2.53%, *p* < 0.01), and increased inpatient mortality (15.28% vs. 5.30% vs. 2.48%; *p* < 0.01) compared to mild–moderate constipation and no constipation. Constipation, regardless of severity, was associated with increased 90-day readmission (30.43% vs. 23.83% vs. 13.64%, *p*< 0.01) and increased 90-day mortality (19.44% vs. 17.80% vs. 5.45%, *p* < 0.01).

In the multivariate regression analysis, anemia (OR: 3.20; 95% CI: 1.32–7.77; *p* = 0.01), atrial fibrillation (OR: 4.11; 95% CI: 1.41–12.00; *p* = 0.01), hypothyroidism (OR: 7.67; 95% CI: 2.37–24.88; *p* < 0.01), and heart failure (OR: 2.48; 95% CI: 1.03–5.98; *p* = 0.04) emerged as independent predictors of ICU/HDU admission. Anemia (OR: 29.94; 95% CI: 3.30–271.72; *p* < 0.01), hypothyroidism (OR: 8.30; 95% CI: 1.69–41.78; *p* < 0.01), and the use of laxatives (OR: 3.89; 95% CI: 1.49–10.11; *p* < 0.01) were identified as independent predictors associated with inpatient mortality. Furthermore, diabetes mellitus (OR: 2.25; 95% CI: 1.34–3.77; *p* < 0.01), chronic obstructive pulmonary disease (OR: 2.65; 95% CI: 1.13–6.23; *p* = 0.03), and frailty score (OR: 1.41; 95% CI: 1.06–1.86; *p* = 0.02) were noted as independent predictors of 90-day hospital readmission. In addition, anemia (OR: 2.39; 95% CI: 1.28–4.48; *p* < 0.01) and frailty (1.53; 95% CI: 1.06–2.23; *p* = 0.03) were independent predictors of 90-day mortality (Table 4).

Among patients with constipation (*n* = 336), only 114 (33.92%) received laxative treatment, with osmotic laxatives, primarily lactulose, being the main type used. Laxative treatment was more common among older patients (*p* < 0.01) and those with higher CAS scores (*p* < 0.01). In constipated patients treated with laxatives, the median CAS score improved (from 6 to 4), although it remained significantly higher than in those not treated for constipation. Regarding health outcomes, more critically ill patients—as indicated by ICU/HDU admission, cardiopulmonary arrest, and longer hospital stays—were treated with laxatives for constipation. There were no differences in other healthcare outcomes between constipated patients treated with laxatives and those who did not receive laxatives (Table 5).

## 4. Discussion

This study represents the first comprehensive prospective study to evaluate constipation’s occurrence, treatment, and related health outcomes in hospitalized patients in medical wards. Key hospitalization metrics were systematically examined, such as LOS, ICU/HDU transfer, and inpatient mortality. Furthermore, patient follow-up extended to 90 days, focusing on mortality and hospital readmission.

The study population represents the diverse group of patients typically admitted to general medicine, characterized by a significant burden of medical conditions, increased frailty, and a high risk of inpatient deterioration and mortality. The prevalence of constipation was significantly high but fell within the range reported in prior studies on hospitalized patients [4,6].

We demonstrated that constipation was linked to prolonged hospital stays, increased inpatient mortality, and a higher need for ICU care. The nature of the association between constipation and worse health outcomes remains unclear, as constipation may be a result of severe illness induced by factors such as dehydration, hypotension, and decreased gut motility caused by the use of multiple medications, including sedative agents [17]. In addition, this association may be explained by shock, which requires vasopressors and results in selective intestinal ischemia. Shock itself is correlated with reduced gut motility, leading to intestinal atony and functional ileus. Consequently, this leads to diminished gut motility and gut hypoxia, collectively contributing to an increased incidence of constipation [17]. Conversely, constipation acts as a risk factor for intra-abdominal hypertension, potentially causing organ dysfunction and exacerbating overall poor health outcomes for inpatients. Patients with constipation are also at an increased risk of bacterial infections due to fecal stasis. These infections can extend hospital stays and worsen overall health outcomes [17,23].

Severe constipation was also associated with an increased prevalence of delirium, consistent with findings from previous studies [6,24]. It remains unclear whether constipation and delirium result from common causative factors or whether delirium leads to constipation or vice versa.

In addition to short-term health outcomes, consequences of constipation extend beyond the hospital stay, and we have found that constipation was associated with an increased risk of 90-day mortality and hospital readmission. In the outpatient setting, a large nationwide cohort of >3 million patients from the USA followed up for more than 6 years found that constipation and laxative use were associated with a higher risk of ischemic heart disease, ischemic stroke, and increased all-cause mortality, independently of known cardiovascular risk factors [25]. In another nationwide study from Denmark involving 83,239 participants, patients were followed up for up to 10 years, revealing an association between constipation and an increased risk of all cardiovascular outcomes [26]. Several mechanisms were proposed to explain this association. Reduced intestinal motility in individuals with constipation has the potential to disrupt the balance of gut microbiota. Recent evidence from large clinical cohorts consistently highlights the contributory role of gut microbiota in the progression of atherosclerosis and cardiovascular diseases [26,27]. Prolonged transit time in constipated patients may facilitate the translocation of pro-inflammatory cytokines from gut bacteria, leading to increased inflammatory responses and oxidative stress [26]. Consequently, individuals with constipation may sustain a chronic state of systemic low-grade inflammation, accelerating the onset of atherosclerosis [28]. This study did not show significant association with either cardiac or thrombotic (i.e., stroke, pulmonary embolism, bowel ischemia) causes of mortality in those constipated.

We demonstrated that constipation was associated with prescribing osmotic laxatives, which is in line with the reported literature [8,25]. Laxatives were administered to only 33.92% of patients with constipation in our cohort. Our findings indicate that older patients, higher CAS scores, longer LOS, and more critically sick patients were more likely to receive laxatives to treat constipation compared to other patients with constipation, which is consistent with previous studies [8,29]. The multivariate regression analysis in our study revealed that the use of laxatives was an independent predictor of inpatient mortality. This finding prompts a question as to whether it reflects the side effects of laxatives or is influenced by patient characteristics, such as requiring more critical care, resulting in longer hospital stays and increased laxative use. It is crucial to interpret this finding cautiously, especially given that only 33.92% of patients with constipation were treated with laxatives. We recommend the conduction of a rigorous study to investigate the causality between constipation, laxative use, and health outcomes among medically admitted patients.

In our setting, osmotic laxatives were used for almost all treated patients; the use of osmotic laxatives is considered the drug of choice for treating transient and chronic constipation, while the bulk-forming agents come first in line due to their safety and mechanism of action [6]. However, they require good fluid intake, which might be inappropriate for most patients during hospitalization, for which osmotic laxatives are widely used [30,31,32].

This study has several strengths, including its prospective design, the utilization of a well-validated score to diagnose constipation, a focus on patients hospitalized in medical wards, and a comprehensive assessment of hospitalization outcomes with a 90-day follow-up.

Being conducted at a single center, this study has inherent limitations. Additionally, as an observational study, we have identified associations between constipation and adverse health outcomes; however, establishing causality is not feasible. Conducting a randomized interventional trial is likely to improve our comprehension of the interplay between constipation, the utilization of laxatives, and negative health outcomes.

## 5. Conclusions

Constipation was associated with several poor health outcomes among medically hospitalized patients. Most patients did not receive laxatives for constipation. Older patients, prolonged LOS, and critical illness were associated with increased laxative use among patients with constipation. It is important to interpret our results with caution; therefore, we believe that a randomized controlled trial will help enhance our understanding of the interaction between constipation, treatment, and poor health outcomes.

## Figures and Tables

**Table 1:** Patients’ characteristics, comorbidities, and primary diagnoses (*n* = 538).

Characteristic, All (*n* = 538)	*n* (%) Unless Specified Otherwise
Age (IQR), years	59 (42–72)
Female	267 (49.63%)
Hypertension	282 (52.42%)
Diabetes mellitus (DM)	225 (41.82%)
Liver cirrhosis	15 (2.79%)
Chronic kidney disease (CKD)	73 (13.57%)
Heart failure (HF)	100 (18.59%)
Atrial fibrillation (AF)	33 (6.13%)
Chronic obstructive pulmonary disease (COPD)	30 (5.58%)
Hypothyroidism	21 (3.90%)
Anaemia	273 (50.74%)
Dementia	21 (3.9%)
**Frailty Class**	
Independent	288 (53.53%)
Partial dependency	148 (27.51%)
Full dependency	102 (18.96%)
**Diagnosis Classified according to the International Classification of Diseases,** **10th Revision (ICD 10)**	
Certain infectious and parasitic diseases (A00-B99)	44 (8.18%)
Haematological diseases (D50-D89)	18 (3.35%)
Endocrinologic and metabolic diseases (E00-E90)	50 (9.29%)
Mental and behavioural disorders (F00-F99)	16 (2.97%)
Diseases of nervous system (G00-G99)	34 (6.32%)
Diseases of circulatory system (I00-I99)	94 (17.47%)
Diseases of respiratory system (J00-J99)	88 (16.36%)
Diseases of digestive system (K00-K93)	49 (9.11%)
Diseases of genitourinary system (N00-N99)	54 (10.04%)
Others	91 (16.91%)

**Table 2:** Incidence of constipation, severity of constipation, and treatment.

Characteristic, All (*n* = 538)	*n* (%) Unless Specified Otherwise
**Constipated status** *n* (%) Patients with constipation/ hospitalized patients	
Constipation at day 0	306/538 (56.88%)
Constipation at day 3	185/303 (61.06%)
Constipation at day 7	81/128 (63.28%)
Constipation at day 14	29/41 (70.73%)
Constipation at day 21	16/21 (76.19%)
Constipation at day 28	9/14 (64.29%)
Overall constipation (cumulative incidence)	336/538 (62.45%)
**Constipation Severity**	
No constipation	202 (37.55%)
Mild–moderate	264 (49.07%)
Severe	72 (13.38%)
Laxatives	134 (24.91%)
Bulk-forming laxatives	0 (0%)
Osmotic laxatives	134 (24.91%)
Stimulant laxatives	0 (0%)
Stool softeners	0 (0%)

**Table 3:** Health outcomes clustered according to constipation status (*n* = 538).

Health Outcomes, *n* (%) Unless Specified Otherwise	All *n* = 538 (100%)	No Constipation *n* = 202 (37.55%)	Mild–Moderate Constipation *n* = 264 (49.07%)	Severe Constipation *n* = 72 (13.38%)	*p* Value
Encephalopathy/delirium	29 (5.39%)	7 (3.47%)	14 (5.30%)	8 (11.11%)	0.048
Depression	14 (2.60%)	4 (1.98%)	8 (3.03%)	2 (2.78%)	0.776
Vomiting	25 (4.65%)	5 (2.48%)	17 (6.44%)	3 (4.17%)	0.129
Aspiration	16 (2.97%)	3 (1.49%)	11 (4.17%)	2 (2.78%)	0.239
Anal fissure	5 (0.93%)	2 (0.99%)	1 (0.38%)	2 (2.78%)	0.170
Hemorrhoids	7 (1.3%)	2 (0.99%)	3 (1.14%)	2 (2.78%)	0.489
Urinary retention	17 (3.16%)	3 (1.49%)	10 (3.79%)	4 (5.56%)	0.170
Intensive care unit/high dependency admission	36 (6.69%)	8 (3.96%)	16 (6.06%)	12 (16.67%)	<0.01
Cardiopulmonary arrest	31 (5.92%)	5 (2.53%)	15 (5.86%)	11 (15.71%)	<0.01
Inpatient mortality	30 (5.58%)	5 (2.48%)	14 (5.30%)	11 (15.28%)	<0.01
Length of hospital stay (IQR)	4 (2–8)	3 (2–5)	5 (2–8)	6 (4–12)	<0.01
90-day readmission	109 (20.84%)	27 (13.64%)	61 (23.83%)	21 (30.43%)	<0.01
90-day mortality	72 (13.38%)	11 (5.45%)	47 (17.80%)	14 (19.44%)	<0.01

**Table 4:** Summary of multivariate regression analysis to identify factors associated with major health outcomes for patient with constipation and without (n = 538).

Outcome/Factors	Adjusted Odds Ratio	95% CI	*p*-Value
**ICU/HDU Admission**			
Anaemia	3.20	1.32–7.77	0.01
Atrial fibrillation	4.11	1.41–12.00	0.01
Hypothyroidism	7.67	2.37–24.88	<0.01
Heart failure	2.48	1.03–5.98	0.04
**Inpatient Mortality**			
Anaemia	29.94	3.30–271.72	<0.01
Hypothyroid	8.30	1.69–41.78	<0.01
Use of laxatives	3.89	1.49–10.11	<0.01
**90-day Readmission**			
Anaemia	1.69	1.03–2.76	0.04
Hypertension	0.46	0.26–0.82	
Diabetes mellitus	2.25	1.34–3.77	<0.01
Chronic obstructive pulmonary disease	2.65	1.13–6.23	0.03
Frailty score	1.41	1.06–1.86	0.02
**90-day Mortality**			
Anaemia	2.39	1.28–4.48	<0.01
Frailty score	1.53	1.06–2.23	0.03

Binary logistic regression analysis incorporated relevant data, such as age, gender, comorbidities, frailty score, length of hospital stay, and constipation-related information.

**Table 5:** Health outcomes in patients with constipation who received laxatives compared to patients who did not receive laxatives (*n* = 538).

Characters and Health Outcomes, *n (%)* Unless Specified Otherwise	Overall Constipated 336 (100%)	No Laxatives 222 (66.07%)	Laxatives 114 (33.93%)	*p* Value
Age (IQR)	64 (46–75)	54 (37–69)	70 (59–77)	<0.01
Female	173 (51.49%)	110 (49.55%)	63 (55.26)	0.321
CAS’s highest score during hospitalization, IQR	4 (3–6)	2 (0–3)	6 (3–7)	<0.01
CAS score before discharge, IQR	3 (2–5)	2 (0–3)	4 (2–6)	<0.01
Encephalopathy/delirium	22 (6.55%)	12 (5.41%)	10 (8.77%)	0.238
Depression	10 (2.98%)	5 (2.25%)	5 (4.39%)	0.276
Vomiting	20 (5.95%)	14 (6.31%)	6 (5.26%)	0.702
Aspiration	13 (3.87%)	10 (4.5%)	3 (2.63%)	0.399
Anal fissure	3 (0.89%)	1 (0.45%)	2 (1.75%)	0.229
Hemorrhoids	5 (1.49%)	3 (1.35%)	2 (1.75%)	0.773
Urinary retention	14 (4.17%)	6 (2.70%)	8 (7.02%)	0.061
Intensive care unit/high dependency admission	28 (8.33%)	12 (5.41%)	16 (14.04%)	<0.01
Cardiopulmonary arrest	26 (7.74%)	9 (4.17)	17 (15.45%)	<0.01
Inpatient mortality	25 (7.44%)	8 (3.60%)	17 (14.91%)	<0.01
Length of hospital stay (IQR)	5 (3–9)	3 (2–7)	6 (4–12)	<0.01
90-day readmission	82 (24.40%)	52 (24.07%)	30 (27.52%)	0.499
90-day mortality	61 (18.15%)	40 (18.02%)	21 (18.42%)	0.928

CAS: Constipation Assessment Scale.

## Data Availability

The data presented in this study are available on request from the corresponding author. The data are not publicly available due to patient-related privacy issues.
